# Effect of perioperative aspirin continuation on bleeding after pneumonectomy

**DOI:** 10.1111/1759-7714.14846

**Published:** 2023-03-13

**Authors:** Qirui Chen, Yan Liu, Yi Liu, Ying Ji, Xin Ye, Xin Li, Yili Fu, Jinbai Miao, Shengcai Hou, Bin Hu

**Affiliations:** ^1^ Department of Thoracic Surgery, Beijing Institute of Respiratory Medicine and Beijing Chao‐Yang Hospital Capital Medical University Beijing China; ^2^ Department of Respiratory and Critical Care Medicine, Beijing Institute of Respiratory Medicine and Beijing Chao‐Yang Hospital Capital Medical University Beijing China

**Keywords:** antiplatelet therapy, aspirin, bleeding, pneumonectomy

## Abstract

**Background:**

To investigate the effect of continuous oral aspirin in perioperative period on bleeding in pneumonectomy.

**Methods:**

A total of 170 patients who underwent pneumonectomy in our hospital from March 2021 to March 2022 were selected as the study objects. All patients took oral aspirin before surgery and did not take other antiplatelet agent or anticoagulants at the same time. The continuation group included 85 cases and continued to take aspirin 100 mg/day during the perioperative period, and the interruption group included 85 cases who stopped aspirin for 7 days before surgery and 3 days after surgery, without bridging therapy. The intraoperative blood loss, operation time, conversion to thoracotomy rate, postoperative bleeding rate, blood transfusion rate, thrombotic events, postoperative drainage volume, length of hospital stay, and total hospital cost of the two groups were compared.

**Results:**

There were no statistically significant differences in intraoperative blood loss, operative time, rate of conversion to open, postoperative drainage, hospital stay, and cost between the two groups (*p* > 0.05), and there were no reoperations due to bleeding between the two groups.

**Conclusions:**

Aspirin should be continued throughout the perioperative period in all high‐risk patients requiring pneumonectomy after balancing ischemic‐bleeding risks.

## Graphical abstract

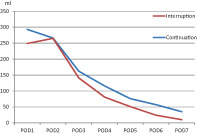

## INTRODUCTION

Aspirin is widely used as an antiplatelet agent for the secondary prevention of atherothrombotic events including myocardial infarction (MI), ischemic stroke and peripheral arterial diseases (PAD). It is sometimes used in combination with the P2Y_12_ inhibitors clopidogrel or ticagrelor, both of which may increase the risk of perioperative bleeding.^**1**^ When patients receiving antiplatelet therapy (APT) require elective surgery, a decision is needed as to whether APT should be ceased, the timing of cessation, and whether bridging therapy is required. The majority of thoracic surgeons have advocated stopping antiplatelet drugs one week before surgery in past decades. Available studies report that the effect of perioperative aspirin continuation on thromboembolic and major bleeding events were varied.^2^, ^3^ With the rapid development of minimally invasive surgical techniques, when patients receiving aspirin are undergoing elective noncardiac surgery, the American Clinical Practice Guidelines suggest aspirin continuation over aspirin interruption.^**4**^ The 2021 Chinese expert collaboration stated that aspirin should not be discontinued during the perioperative period for major tumor surgery and major thoracic surgery for patients with atherosclerotic cardiovascular disease with high or very high risk of ischemic cardiovascular events.^**5**^ However, there have been few studies on aspirin continuation during the perioperative period of pneumonectomy, therefore, we sought to investigate if perioperative aspirin interruption is required.

## METHODS

A total of 170 adult patients who received pneumonectomy in our hospital from March 2021 to March 2022 met the study inclusion criteria and were considered for the analysis. All patients without bleeding disorders took aspirin 100 mg/day for more than one month before surgery, and no other antiplatelet agent or anticoagulants were taken at the same time. The continuation group included 85 cases and continued to take aspirin 100 mg/day during the perioperative period. The interruption group included 85 cases who stopped aspirin for 7 days before surgery and 3 days after surgery, without bridging therapy. The operation was performed under general anesthesia with double‐lumen tracheal intubation and video‐assisted thoracic surgery (VATS) with two ports. Intraoperative hemostasis was sufficient and blood loss and operation time was recorded. A thoracic drainage tube was routinely placed through the observation port. If there was no air leakage, the drainage was clear and less than 300 ml/day postoperatively, the tube would be removed and the patient discharged then or the next day.

### Statistical analysis

Statistical analyses were conducted using SPSS19.0 (IBM). Continuous variables are presented as means ± standard deviations (SD) or medians and interquartile ranges (IQR) and were analyzed using an independent‐sample *t* test or Mann–Whitney U test for variables with and without normal distribution, respectively. Categorical variables are presented as numbers and percentages, and compared using chi‐square test or Fisher's exact test. Two‐sided tests were performed, and *p* < 0.05 was considered statistically significant.

## RESULTS

### Comparison of baseline clinical characteristics

Among the 170 subjects who received pneumonectomy, VATS was performed successfully in 157 patients; four cases were converted into open surgery owing to adhesion or hemorrhage, and there were nine cases of scheduled thoracotomy. There was no significant difference in age, sex, body mass index (BMI) or duration of aspirin between the two groups (*p* > 0.05). In terms of surgical methods, the proportion of segmentectomy in the aspirin continuation group was a little higher than that in the aspirin interruption group (*p* = 0.025). In terms of comorbidity, the patients in the continuation group had more MI (*p* = 0.029), while those in the interruption group had more hyperlipidemia (*p* = 0.044) (Table 1).

**TABLE 1:** Baseline characteristics.

Variables	Continuation *n* = 85	Interruption *n* = 85	Test value	*p*‐value
Age (y), mean ± SD	67.68 ± 7.397	66.28 ± 7.106	*t* = 1.258	0.210^a^
Female sex, *n* (%)	35(41.2%)	32(37.6%)	0.222	0.638^b^
BMI (kg/m^2^), mean ± SD	25.22 ± 3.093	25.31 ± 3.284	*t* = −0.188	0.851^a^
Operation, *n* (%)				
VATS lobectomy	39(45.9%)	44(51.8%)	0.589	0.443^b^
VATS segmentectomy	20(23.5%)	9(10.6%)	5.031	0.025^b^
VATS wedge resection	21(24.7%)	24(28.2%)	0.272	0.602^b^
Open pneumonectomy	5(5.9%)	8(9.4%)	0.750	0.387^b^
Pleural adhesions, *n* (%)	13(15.3%)	7(8.2%)	2.040	0.153^b^
Lymph node dissection, *n* (%)	42(49.4%)	48(56.5%)	0.850	0.357^b^
Malignant, *n* (%)	79(92.9%)	81(95.3%)	0.425	0.514^b^
Duration of aspirin, *n* (%)	12(3,60)	36(6,84)	*Z* = −1.749	0.080^c^
Smoking, *n* (%)	36(42.4%)	33(38.8%)	0.220	0.639^b^
Comorbidity, *n* (%)				
Hypertension	61(71.8%)	58(68.2%)	0.252	0.616^b^
DM	38(44.7%)	26(30.6)	3.608	0.057^b^
Hyperlipemia	30(35.3%)	43(50.6%)	4.057	0.044^b^
CAD	60(70.6%)	50(58.8%)	2.576	0.109^b^
Arrhythmology	6(7.1%)	1(1.2%)	2.384	0.123^b^
Prior MI	9(10.6%)	2(2.4%)	4.763	0.029^b^
Prior ischemic stroke	15(17.6%)	25(29.4%)	3.269	0.071^b^
PAD	11(12.9%)	7(8.2%)	0.994	0.319^b^

*Note*: Statistical methods: ^a^Independent‐sample *t* test. ^b^Chi‐square test. ^c^Mann–Whitney U test.

Abbreviations: BMI, body mass index; CAD, coronary artery disease; DM, diabetes mellitus; MI, myocardial infarction; PAD, peripheral arterial disease; SD, standard deviation; VATS, video‐assisted thoracic surgery.

### Comparison of operation characteristics

There were no significant differences in operation duration, intraoperative blood loss, intraoperative blood transfusion, and conversion to open rate between the two groups (*p* > 0.05). No intraoperative hemostatic drugs were used for all cases (Table 2).

**TABLE 2:** Operation characteristics.

Variables	Continuation *n* = 85	Interruption *n* = 85	Test value	*p*‐value
Duration of operation (min), median (IQR)	120 (90,160)	120 (87.5,150)	*Z* = −1.180	0.238^a^
Blood loss (ml), median (IQR)	50 (20,100)	50 (20,100)	*Z* = −1.314	0.189^a^
Blood transfusion, *n* (%)	0 (0)	2 (2.4%)	0.506	0.477^b^
Conversion to open, *n* (%)	1 (1.2%)	3 (3.5%)	0.256	0.613^b^

*Note*: Statistical methods: ^a^Mann–Whitney U test. ^b^Chi‐square test.

Abbreviation: IQR, interquartile range.

### Tendency of thoracic daily drainage

**FIGURE 1:**
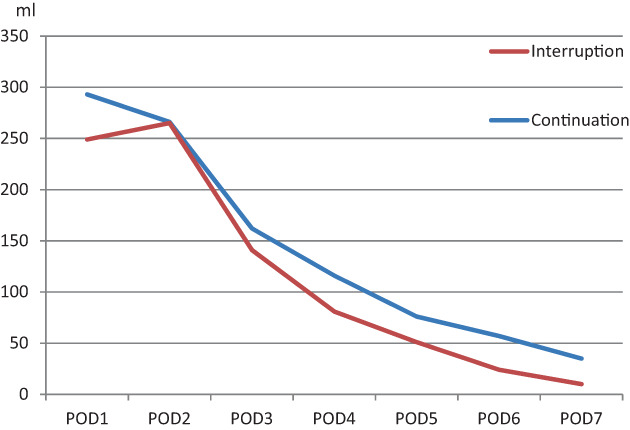
Daily thoracic drainage.

Thoracic drainage was relatively higher on post operation day (POD) 1 and POD 2, although the average level was below 300 ml/day. The time of postoperative drainage was similar between the two groups (*p* = 0.150), and there was no statistical difference in the total amount of drainage (*p* = 0.062) (Figure 1 and Table 3).

**TABLE 3:** Postoperative clinical characteristics.

Variables	Continuation *n* = 85	Interruption *n* = 85	Test value	*p*‐value
Drainage time (day), median (IQR)	4 (3,5)	3 (3,5)	*Z* = −1.440	0.150^a^
Total drainage volume (ml), mean ± SD	1005.82 ± 751.301	820.20 ± 516.217	*t* = 1.877	0.062^b^
Postoperative hospital stay (days), median (IQR)	4 (3,6)	4 (3,6)	*Z* = −0.239	0.811^a^
POD2 Hb decrease (g/dl), median (IQR)	8 (1,16)	6 (0,12)	*Z* = −2.085	0.037^a^
Postoperative blood transfusion, *n* (%)	0 (0)	2 (2.4%)	0.506	0.477^c^
Postoperative MI, *n* (%)	0 (0)	1 (1.2%)		1.000^d^
Postoperative stroke, *n* (%)	0 (0)	1 (1.2%)		1.000^d^
Postoperative VTE, *n* (%)	1 (1.2%)	6 (7.1%)	2.384	0.123^c^
Postoperative mortality, *n* (%)	0 (0)	1 (1.2%)		1.000^d^
Hospital cost (10 000 yuan), mean ± SD	5.86 ± 1.529	5.56 ± 1.475	*t* = 1.288	0.200^b^

*Note*: Statistical methods: ^a^Mann–Whitney U test. ^b^Independent‐sample *t* test. ^c^Chi‐square test. ^d^Fisher's exact test.

Abbreviations: IQR, interquartile range; SD, standard deviation; POD, post operation day; MI, myocardial infarction; VTE, venous thromboembolism.

### Comparison of postoperative clinical characteristics

None of the patients underwent reoperation due to bleeding. There was no intra‐ or postoperative blood transfusion in the aspirin continuation group. There was significant hemoglobin (HB) decline in the aspirin continuation group compared with the aspirin interruption group on POD2, but the difference was very small with little clinical significance and no life‐threatening bleeding or significant hypotension. There was no significant difference in postoperative drainage time, drainage volume, postoperative hospital stay, and cost between the two groups (*p* > 0.05). There was no significant difference in the incidence of arterial thromboembolism (ATE) (*p* = 1.000) and venous thromboembolism (VTE) (*p* = 0.123) between the two groups. One patient in the aspirin interruption group died of bronchopleural fistula following right middle and upper lobectomy (Table 3).

## DISCUSSION

Platelets play a key role in the development of atherothrombosis and thrombotic events. Aspirin suppresses the production of thromboxane‐A_2_ through irreversible inhibition of cyclooxygenase‐1 that is expressed in platelet and megakaryocyte, and the inhibitory effect lasts the lifespan of the platelet,^**6**^ thus aspirin can inhibit platelet aggregation and play an antithrombotic role. Aspirin can effectively reduce the risk of death in patients with coronary artery disease (CAD), and reduce the occurrence of cardiac adverse events such as MI and stroke.^**7**^ Low dose aspirin use is therefore recommended in the primary prevention of cardiovascular events in certain high‐risk patients by most countries, and also commonly prescribed in patients with stable CAD or chronic symptomatic PAD, and prescribed for the secondary long‐term prevention of stroke and transient ischemic attack.^**8**^

About one‐quarter of patients undergoing percutaneous coronary intervention (PCI) require noncardiac surgery in the subsequent 2 years.^**9**^ Premature cessation of APT is associated with an increased incidence of thrombotic complications and cardiovascular events.^**10**^ Platelet aggregation and rebound phenomenon may occur following APT cessation, and thrombosis complications are often acute and severe, even resulting in death. Perioperative aspirin therapy in patients with previous PCI was associated with a significant decrease in the risk of death and nonfatal MI, without increasing the risk of bleeding.^11^, ^12^, ^13^ In addition, aspirin reduces the risk of VTE by at least one third throughout a period of increased risk.^**14**^

MI and major bleeding are leading attributable causes of mortality in the 30 days after noncardiac surgery,^**15**^ and most adverse thrombotic and bleeding complications may relate to perioperative antithrombotic management, which requires balancing an individual's risk of thrombotic complications (ATE and VTE) against anticipated bleeding risk in light of each patient's clinical characteristics and settings. Except for the proinflammatory and hypercoagulable states induced by the anesthesia and operation, the bleeding itself can act as a precipitating factor for ischemia, and the two conditions can frequently coexist. The risk of thrombotic events is heightened in patients who experience perioperative bleeding events.^**16**^

The types of surgery affect bleeding and thromboembolic risk. Aspirin should be continued throughout the perioperative period in all patients requiring elective cardiac surgery.^**8**^ Perioperative aspirin continuation was not associated with an increased rate of major bleeding following general or abdominal surgery,^**17**^ spinal surgery,^**18**^ and intracranial tumor surgery.^**19**^ The American Clinical Practice Guidelines suggest that patients undergoing dental, dermatological or ophthalmological procedures should continue aspirin and the P2Y_12_ inhibitor should be interrupted.^**4**^ According to the European guidelines, P2Y_12_ inhibitors can be stopped if aspirin can be maintained throughout the perioperative period of elective surgery after one month following PCI.^**1**^ DAPT seems to increase the risk of both major adverse cardiac events and bleeding while monotherapy is comparable with no APT.^**12**^

Generally, major thoracic surgery, cancer surgery, especially solid tumor resection and any major operation (procedure duration >45 min) are regarded as high‐bleed risk surgery,^**20**^ so temporary aspirin interruption is very common owing to bleeding concerns in the last decades. However, in recent years, the incidence of lung cancer has been increasing rapidly and the proportion of anatomical sublobar resection has been increasing. The scope of lymph node dissection has also been individualized, VATS pneumonectomy has become mainstream and mature, and the procedure is more common among elderly patients receiving APT, who are usually at high risk of thromboembolism, so the risk–benefit balance of APT should be reassessed.

Our study aims to deliver individualized, patient‐centered precision APT with the intent of minimizing perioperative thromboembolism and major bleeding. The risk of major bleeding complications is directly related to the operation techniques. Uninterrupted perioperative aspirin requires a standardized procedure and thorough hemostasis, especially for patients with extensive adhesions, the surgical field must be carefully checked for active bleeding and repeated local hemostasis before closing the incision is essential. In our study, almost all the patients who received oral aspirin had a history of hypertension, CAD, DM, MI, or stroke. There were no significant differences in intraoperative bleeding, operation time, intraoperative blood transfusion rate and rate of conversion to open between the two groups. The reason for conversions in our study was lymph node severe adhesion or accidental injury to the pulmonary artery, which were not directly related to the continuation of aspirin.

Multifaceted elements should be considered before deciding on a perioperative APT strategy. A consensus decision about preoperative APT based on a referral system among physicians, surgeons, and anesthesiologists is favorable for most cases. Evaluation of the procedure‐related bleeding risk also includes the type of anesthetic technique selected. The risk of spinal hematoma related to aspirin appears very low, so aspirin is not a contraindication to neuraxial anesthesia if the benefit–risk ratio is favorable.^**8**^ In most clinical situations, continuous aspirin provided benefit that outweighed the bleeding risk, so most guidelines recommend that aspirin is continued perioperatively.^**3**^ However, aspirin cessation should be considered when it comes to the procedures associated with a very high risk of hemorrhagic complications, such as a surgical procedure for pulmonary abscess with empyema or pulmonary resection for destructive pneumonophthisis, for which the American Clinical Practice Guidelines suggest stopping aspirin ≤7 days before the surgery, and resuming an antiplatelet agent ≤24 h after the surgery.^**4**^

There was a lack of consensus in a previous study with regard to the use of bridging therapy with heparin or intravenous antiplatelet agents during perioperative antiplatelet cessation, as the bridging therapy demonstrated no clear benefits.^**4**^ The perioperative use of unfractionated heparin or low molecular weight heparin (LMWH) is associated with an increased risk of bleeding,^**21**^ and early postoperative full doses of LMWH are prone to major bleeding, even if there is no obvious sign of bleeding.^**4**^ As a result, heparin bridging should be administered in a manner which minimizes the risk of major bleeding. However, for high‐risk patients within one month following PCI, an intravenous antiplatelet agent should be considered,^**22**^ and in patients who are considered at high risk for postoperative VTE, low‐dose LMWH can be used for the first 24–72 h post‐operation, with full dose LMWH resumed 2–3 days post‐operation.^**4**^

In our study, there was a hemorrhagic complication only once in the aspirin continuation group, but we found that the drainage tube port was not tightly stitched and bleeding was stopped after an additional stitch without transfusion, and aspirin continued to be taken from then with 2 days cessation. As to the severe bleeding complication associated with the antiplatelet agent, just like hemorrhagic shock, intracranial and gastrointestinal hemorrhage, if hemostatic measures are not sufficient to stop the bleeding, transfusion of sufficient normal platelets should enable hemostasis by replacing the inhibited platelets.^**23**^ Guidelines also propose platelet function testing prior to transfusion when possible.^**24**^

This was a single‐center study with a relatively small sample size. As for the influence of perioperative aspirin continuation on ATE and VTE, further studies are required in this respect. In addition, postoperative administration of low‐dose LMWH in some patients may have had an impact on the results of the study. As far as aspirin continuation in other thoracic operation scenarios except for pneumonectomy, further investigation is warranted.

In conclusion, aspirin continuation during the perioperative period of pneumonectomy did not affect the incidence of blood transfusion, although it had certain influence on the level of postoperative HB. Aspirin continuation did not increase the postoperative drainage time, hospital stay, blood transfusion, postoperative mortality, and hospitalization cost, which is safe and feasible, so aspirin 100 mg/day should be routinely maintained for high‐risk patients after balancing ischemic‐bleeding risks.

## AUTHOR CONTRIBUTIONS

Conception and design: Qirui Chen, Bin Hu. Administrative support: Shengcai Hou. Provision of study materials or patients: Xin Ye, Xin Li, Yili Fu. Collection and assembly of data: Yi Liu, Ying Ji. Data analysis and interpretation: Yan Liu, Jinbai Miao. Manuscript writing: Qirui Chen. Final approval of manuscript: All authors.

## CONFLICT OF INTEREST STATEMENT

The authors declare that the research was conducted in the absence of any commercial or financial relationships that could be construed as a potential conflict of interest.
